# Some Practical Considerations in Chronic Heart Disease*Read at meeting of Alumni Assn., Univ, of Buffalo, June, 1917.

**Published:** 1917-09

**Authors:** Arthur J. Patek

**Affiliations:** Milwaukee, Wis.


### BUFFALO MEDICAL JOURNAL

Yearly Volume 73 SEPTEMBER, 1917 Number 2

### ORIGINAL ARTICLES

The right is reserved to decline papers not dealing with practical medical and surgical subjects, and such as might offend or fail to interest readers. Contributors are solely responsible for opinions, methods of expression and revision of proof.

### Some Practical Considerations in Chronic Heart Disease.*

ARTHUR J. PATEK, A. B., M. D., Milwaukee, Wis.

In submitting a consideration of some facts that have a practical bearing upon our conception of disease of the heart, I will preface my remarks with an apology for their brevity and necessary incompleteness. In selecting but a few facts for presentation—without an effort at systematization—I am aware that there must be and are many others of equal—perhaps even greater—importance and value from a practical and therapeutic standpoint, and which could as well be included, or even substituted. But even these few have a real bearing upon a successful conception of the disease, and I trust I may convey them in a manner to emphasize their significance.

Before doing so, however, I would digress a moment in order to call attention to a popular misnomer (emphasized by Stengel): the term "chronic endocarditis" is commonly used to describe either a compensated valvular defect as evidenced by heart murmurs, or a broken cardiac compensation with heart murmurs. The term is wrongly applied in both instances, because valvulitis in the sense of a chronic and progressive illness, does not exist—(save in the more unusual forms of chronic syphilitic infection). The compensated valvular defect, whose presence is demonstrated by the usual signs, is the result of a previous acute valve disease, and constitutes a healed valve deformity, not a continuing—or progressive—affection. Once healed, compensation takes

*Read at meeting of Alumni Assn., Univ. of Buffalo, June, 1917.

place, nor will it ever again—acting alone—give rise to symptoms unless there be an endocarditis engrafted upon it. Therefore it were well if we dismissed the erroneously used term "chronic endocarditis" when referring to defective but healed valves, and, in its place, speak of valvular defects, or simply of murmurs, or single out the particular name and character of valvular abnormality.

The mechanics of the circulation, or its physiology, must be appreciated in order to properly estimate its pathology. The essential elements in the maintenance of normal circulation are: 1. The propelling or driving power of the heart; 2. The condition of the arteries and arterioles; 3. The vasomotor mechanism, particularly in so far is it pertains to the splanchnic circulation, and controls the mass movement of the blood.

1. **The Propelling Power of the Heart.** We may consider it a fundamental fact that the functionating capacity of a heart is measured by its muscle, not its valves, and that upon the integrity of the heart muscle and its ability to adapt itself to varying conditions, rests the big burden of maintaining a proper equilibrium. In order to prove equal to unusual demands which an exacting economy makes, the heart muscle is compelled to, and does, become hypertrophied. In this manner compensation is developed in healing valvular lesions, and in cases in which excessive strain—through unusual physical effort—is put upon the heart. The opinion is often expressed that the body harboring such a heart is as safely insured against damage from this source as is one whose heart's integrity has never been questioned. This is an erroneous idea. True, the hypertrophy that has developed does not necessarily reduce the heart's ability to take up and satisfactorily carry the burden of the body's physical activity, under ordinary conditions. There is a limit to hypertrophy, however, and even when it is considerable the power that can be developed is never so largely in excess of that ordinarily required as in a normal heart. The reserve power of such a heart is reduced, and what Mackenzie calls the "field of response" is therefore limited. When confronted with unusual demands this hypertrophy has frequently proven a blind, and disaster has been the inevitable consequence. Such, per example, is the case of the athlete whose heart, through long education, suits its action to its master's bidding; it becomes hypertrophied, responds to every demand, and makes its owner happy in a consciousness of strength. But an unwonted acute demand, whether in the shape of a physical effort, or a mild or severe infection, or shock, has in many an instance easily broken down this compensation, the first line of defense found defective, the much needed mainstay been proven a disastrous disappointment.

We must at all times bear in mind that, inasmuch as it is the heart muscle's hypertrophy that makes possible a symptomatic recovery from a valvular defect, this equilibrium can be maintained only so long as the muscle's integrity is maintained. Therefore, when symptoms of broken compensation arise in such a case, they do so as a result of muscle—not valve—decompensation. I believe this distinction worthy of emphasis because we have been too prone to consider the valve defect as the object of our solicitude, rather than the muscle disabled as a result of that defect. While this analysis may seem to have but an academic value, in reality its proper recognition has a direct bearing upon our therapy, and merits greater attention in text book essays than has hitherto been accorded it.

2. **Arterial or Arteriole Disease** is very commonly the barrier upon which the heart vents itself in vain, and to which effort, having spent itself, it finally succumbs. We are, in the one instance, dealing with a heart compensated against a valvular defect acquired in early life or in adolescence, and permitting its possessor the enjoyment of perfect health and activity. But with the advent of middle life arterio-sclerotic changes supervene, and the situation at once becomes critical; the inelasticity of the blood vessels offers greater resistance, the heart is overtaxed, and dilatation gradually develops. Or, we may be dealing with a heart that has had to overcome frequent onslaughts in the nature of splanchnic engorgements or mesenteric sclerosis. And lastly, but most important of all, chronic renal disease gives rise to high tension, arterial degeneration, and eventually cardiac disease, with the same disastrous consequences.

3. **The Vasomotor Mechanism** plays a vital part in the maintenance of vascular equilibrium. The splanchnic circulation being the "largest vascular field in the body," is therefore most concerned in vasomotor influences, and, when disturbed, may affect the circulatory equilibrium. This is a factor in all processes of digestion, in the engorgement which may follow overfeeding, and affects particularly the middle aged and those of sedentary habit. Anginoid attacks are common in these, and especially in the overfed type of individual with large and pendulous abdomen.

**Hypertension.** Allbutt claims that asterial disease is not the cause of elevated blood pressure, but its results; that high pressure cannot be long maintained without arterial strain, and that "arterio-sclerosis results from long persisting high blood pressure from whatsoever origin.'' The causes of high pressure are, in individual cases, obscure. Toxemia, renal disease, gout, and other metabolic errors, possibly adrenalin excretion, and poisons, play a part. In elderly people the blood pressure may be elevated without evidence of renal or other disease. Under the term ''Nephritic Hypertension'' we refer to the cases of chronic Bright's with hypertension. When renal disease does **not** exist, an intoxication of obscure origin is probably the exciting cause producing increased peripheral resistance and elevation of blood pressure. This is the type of ''Essential Hypertension'' which may persist for years with no sign of nephritis or with only occasional slight albumenuria. But these, I think, in many instances, are linked with and develop into the nephritic hypertension cases, and when very persistent—often and finally succumb to a uremia. I am wont to consider all persistent high tension cases as **potential** chronic nephritics.

The fallacy of making Arterio-Sclerosis and Hypertension synonymous terms and conditions, is still common enough to warrant mention. It is to be remembered that there may be marked beading of the arteries without hypertension, and soft arteries with high tension. It is rather difficult to reconcile this with Allbutt's statement that high tension precedes—that is—causes arterio-sclerosis, unless one were willing to concede that primarily high tension existed in all cases, as the result of a toxemia, and that—after arterio-sclerosis had developed—the tension became reduced on the basis of Muenzer's theory of the causation of hypotension in arterio-sclerosis; ''the blood flows through the large blood vessels as does a fluid through an unyielding tube; there being no periodic distension of the vessel wall, the tension is lowered.'' (Goodman, Am. J. Med. Sc. April 1914).

Far more serious than the cases of marked hardening of the superficial arteries—with or without symptoms, are the cases of hypertension in those of soft arteries. In the latter this disease is dangerous, because it is insidious, and does not incapacitate. In this class are men and women of middle life and of portly frame; men of responsibility, women with large families. These patients look healthy, their radials are soft, temporals not hardened; arcus senilis is present, their hearts somewhat enlarged, systolic pressure as high as 200 to 250, second aortic accentuated. Riesman (Am. J. Med. Sc. 1913, p. 487) considers the finding of a systolic aortic murmur in these cases of prognostic import; that it means sclerosis of the aorta and coronaries, and therefore indicates a greater tendency to the development of angina pectoris. As a matter of fact, these high pressure cases—without visible or palpable arterio-sclerosis, are particularly prone to this serious outcome.

**Significance of Systolic and Diastolic Pressure.** The Systolic pressure can best be described as the indicator of the force of the ventricular contraction of the heart; the Diastolic as the indicator of the amount of peripheral resistance in the arterial system. The difference between these two is the Pulse Pressure—the excess force the heart exerts over the peripheral resistance—or the amount of pressure which carries on the circulation—or, put in other words, that part of the heart's energy which produces the distension of the arteries and is recognized as the pulse. The systolic and pulse pressure readings represent myocardial values, the diastolic arterial resistance. Therefore, the systolic reading alone, while of value, is misleading unless checked up with the diastolic.

When may there be considered to exist a normal balance between the systolic and diastolic pressures? According to authorities, the pulse pressure is normally approximately 35% of the systolic, or 50% of the diastolic reading. Marked deviations from these figures suggest a tendency to—or already the presence of—a lack of balance between heart muscle and peripheral circulation. In an effort to properly interpret the clinical significance of these phenomena, W. J. Stone (J. A. M. A., Oct. 1913) has suggested the use of a formula expressed by the pulse pressure divided by the diastolic pressure as indicating the cardiac load and "overload." The more nearly the pulse pressure approaches the diastolic in a case of myocardial disease, the greater is the danger of impending muscle insufficiency. The systolic pressure in this instance is relatively high, and therefore indicates excessive effort on the part of the heart, resulting in overstrain. On the other hand, where the pulse pressure is low, because the diastolic is increased, this excess of peripheral pressure threatens greater danger from cerebral accident.

It is necessary to emphasize this subject because, despite the insistence of recent writers, there is still manifest a great disinclination to deal with any but the systolic reading.

**Shall Hypertension be Treated?** It must be borne in mind that a high blood pressure is such in a relative sense only, and that not all high pressures demand lowering. In fact, a high blood pressure is undoubtedly frequently a compensatory—therefore conservative phenomenon, a measure of defense, not a destructive process, and is part of a balanced system, just as the hypertrophied heart is an expression of balance—an insurance against accident. Therefore, the administration of vasodilators in all cases of high tension is not only an act of folly, but a positively dangerous practice, and in instances may lead to disaster. When it does produce symptoms, it may be reduced to a point where these symptoms cease, and not beyond. Many persons with moderate hypertension have disturbing symptoms, while many with high tension are thoroughly comfortable. Persons of good muscle development and active habit are better able to withstand a hypertension than are the obese and sluggish.

**Symptoms.** Among the **early** symptoms of cardiac distress may be enumerated: dyspnea, cough, palpitation, anginoid pain, slight oedema of the legs, gastro-intestinal disturbance, epistaxis, vertigo, faintness, headache, insomnia, tingling and numbness, ringing in the ears, and nocturnal polyuria. I emphasize these symptoms because they are not the severe ones of cardiac decompensation, and because, being mild, their proper interpretation may, in instances, be overlooked. A complaint of polyuria, especially nocturnal, with severe headache, or eye disturbances, in a patient below middle age with high blood pressure, suggests an attack of uremia as imminent.

Among the symptoms enumerated, I would particularly emphasize that of dyspnoea. It has been stated "that myocardial insufficiency can be expressed in terms of dyspnea; that where there is complete balance between pulmonary and systemic circulation dyspnea is absent on exertion; where incomplete balance, dyspnea occurs upon slight exertion; where the myocardium is insufficient dyspnea occurs even when at rest, and is exacerbated by the slightest exertion, such as talking or eating." Too much emphasis cannot be placed upon this one symptom—as an indicator of returning heart capacity. It is extremely difficult to appeal to a patient in terms that are purely medical, and that consequently have no meaning to him. To warn him against an excess because his pulse is arythmic or too rapid, or because of slight precordial pressure or cough, is frequently an unconvincing argument, and fails to strike home. But by compelling him to recognize that in dyspnea he has an admonishing symptom that is real, that is a tangible flag of distress of which he himself is even more cognizant than his physician. I have found myself able to more easily curb the convalescent's impatience, and guide him to a safer conduct.

**Case History.** In order to present a concrete example illustrating the compensatory nature of high pressure, and the fallacy, as well as danger, of interpreting cardiac conditions by the systolic reading alone, I wish to cite in some detail the case of Mr. H. S., aged 63, who has been under my observation many years. During the past winter he had occasion to visit northern Michigan. A heavy snow-drift stalled his automobile, and he gave a willing hand to his chauffeur in an effort to push the car through the drift. Unsuccessful, he walked to the nearest town—a distance of three miles—for help, and arrived there in an exhausted state, complaining of heart palpitation and dyspnoea. He called a physician who saw him twice, and expressed his satisfaction that the patient's blood pressure (systolic) of 140 showed a good heart despite the arythmia present. A day's rest improved him slightly, and he left for a neighboring city. Symptoms of cardiac distress again arose. Another physician was called, and again favorable comment was made, because of a systolic blood pressure reading of 130. Experiencing no improvement he left for his home where I saw him on the following day. He was exhausted, dyspneic, suffered precordial distress, heart's action was arythmic, systolic pressure 130, diastolic 105, giving a pulse pressure of 25. The systolic pressure soon fell to 110, and with this there were marked arythmia and distress upon the slightest exertion. Improvement in symptoms came with a gradually rising systolic pressure, and when this, after many weeks, finally reached 150, and then 165, the patient felt himself reasonably well, and remains so at present writing.

Now let me trace back this patient's history for a few significant facts. In 1910 a trace of albumen, and a few hyaline casts were found in the urine, and slight dyspnea upon exertion was complained of. The urinary findings disappeared, nor to my knowledge did they reappear during a five year period following, although he doubtless was during all this time a latent nephritic, or—we may say—he was suffering from a potential interstitial nephritis.

He presented himself for examination from time to time, rather from a sense of precaution, then because of symptoms. His systolic pressure fluctuated between 160 and 180, the diastolic between 100 and 120, with usually a reasonably normal pulse pressure. Compensation had thus been fully established, and maintained during a period of more than six years, between the driving power of the heart and the peripheral resistance it had to overcome—in other words—the circulation had become equalized. An obscure infection supervened in 1915, followed by a prostatic abscess. Definite sings of chronic nephritis developed, and later uremic symptoms, from which he eventually recovered completely. He remained well until the occurrence of acute cardiac breakdown described above. The long persistent hypertension suggests this to be a case of essential hypertension only; yet when a complication ensued, uremia developed. And now he is again—symptomatically—purely a case of essential hypertension.

Deduction of the physicians who saw him at this time was erroneous in that they made their systolic reading of between 130 and 140 the basis of the opinion that the heart was functionating well; whereas, had the diastolic reading been made as well, and the reduced pulse pressure noted, an acute lowering of the established equilibrium would have been seen, and the acute cardiac breakdown recognized. The systolic pressure of 130 or lower was wholly inadequate for the actual needs of this man of 63, and my insistence that he would not be considered recovered until it had reached its former level of 160 to 170 proved fully justified by the later development.

I believe that clinical facts warrant the generalization that in an individual 55 to 60 years of age, a systolic pressure reading of 140 or under is suspicious of a weak heart muscle, and may indicate the actual case of indefinite symptoms of distress, irrespective of the heart's sounds and rhythm.

**Treatment.** It would take me too far afield were I to distinguish—for the sake of therapy—carefully between cardio, cardio-renal and cardio-arterio-renal disease, because the general rules that may be laid down apply to all, although careful analysis of the individual case will, of course, determine the particular procedure. Treatment varies as to 1) the Stage of Compensation; and 2) Stage of Decompensation.

In the Stage of Compensation every effort must be bent to avoid an unnecessarily early decompensation. The heart must be safeguarded against overstrain. Moderate, but regulated exercise should be encouraged; overindulgence in food and drink discouraged, and all excesses avoided. The diet should not be too liberal, and if obesity exists, should be curtailed or properly selected. The confirmed arteriosclerotic, that is—cardio-renal patient, should have a low protein diet, and should by a careful regulation of the bowels studiously avoid splanchnic congestion. He is to avoid physical fatigue, but is to indulge in exercise to the limit of his capacity, and must particularly shun anything that may cause undue mental strain. He should preferably have an occupation, but ought to adjust his work so that it can be conducted without unnecessary cardio-vascular strain. Aside from this—hydrotherapy may be useful, and drugs may be symptomatically used.

In the Stage of Decompensation, one's attention is first directed to the very effectual first line of defense provided in the body for this emergency in the **Conservative Action of the Liver and Spleen.** These two organs are capable of enormous distension, and where cardiac over-dilatation threatens, will provide a ready outlet for the blood which would otherwise cause a rapid and fatal overdistension of the heart. The engorgement of the liver, therefore, in cases of decompensation, is not an unwelcome sign. As compensation is again restored this enlargement subsides, and the organ contracts to its normal size. Repeated liver engorgements, if acute, may take place, and recovery follow. With the advent of long standing or permanent decompensation, the hepatic hyperaemia leads to a low grade inflammation, and eventual secondary induration, with resultant permanent enlargement. This passively engorged, sclerotic liver (Stauungs Leber)—once developed—is in itself a reasonably good record of the patient's cardiac history.

Briefly summarized among the measures of relief in broken compensation are:

1. **Rest**—absolute and prolonged—the first essential in every form of decompensation. Later, the heart's response to exercise, and presence or absence of dyspnea, are good indicators of its improved function.

2. **Digitalis,** when given in the usual therapeutic dose, does not increase blood pressure, and may be safely given in all cardiac breakdowns. It will often be found useful in small tonic doses, even when systolic tension is high.

3. **Opium** is of inestimable value, and is indispensable in most cases of broken compensation. When combined with digitalis, the latter often has an effect which it cannot produce acting alone.

4. **Diet** may reduce high pressure and relieve symptoms due to hypertension. In cases of decompensation with dropsy, with a pulse of fair volume, the Karrel-Kur—(1 quart of milk daily in divided doses, without the addition of other food or drink) is frequently followed by a startlingly quick cardiac response, and an early subsidence of the dropsy.

5. **Depletion** by calomel, blue mass, or other cathartics, is often called for, before digitalis can make an impression upon the disabled heart, because the mass movement of blood gives rise to splanchnic engorgement, thus creating an impediment to the circulation.

6. **Venesection** in high tension with symptoms is a remedy of no mean value. An acutely oncoming epistaxis has often prevented a cerebral accident; we can do likewise with our lancet, and should resort to it more frequently than is our custom.

7. **Vasodilators** I place at the lowest rung in this therapeutic ladder, because—while in very common use—their reputation for service has exceeded their efficiency. They are not heart tonics, and are only to be given where high pressure head symptoms are present, or anginoid attacks exist or threaten; here the vasodilators are indispensable and will often act promptly and excellently, but their action does not extend beyond symptomatic relief, and is very evanescent. When nitro-glycerine fails, it may be because too small a dose is given. Several doses, repeated at very brief intervals may give speedy relief, where the usual small dose fails. Thyroid Extract, while frequently not reducing the tension, will give marked symptomatic relief. I have found it particularly useful in allaying distressing tinnitus and dizziness.

8. Other measures—stimulants, sedatives, and diuretics, are useful also, but those mentioned I consider of prime importance, indispensable in cardiac breakdowns, whether of the acute—or cardio-renal type.

It were gratifying did one feel warranted in asserting that high tension patients—if only their pulse pressures are relatively normal—are in no danger of accident. Experience has taught us to fear these cases, more especially because their resistance is constantly below par, and because they are potential nephritics.

I have thus sketched—under a few chapter headings—disconnectedly to be sure—and necessarily merely in rough outline—a few items that are pertinent to the big subject of arterio-cardio-renal disease. Our knowledge of them is still limited, our therapy halting and uncertain, and its results inconclusive. Despite this—good judgment and advice on our part, and an infinite amount of patience and co-operation on the part of the invalid, will add to his comfort and prolong his usefulness.

---

**Examination for Tubercle Bacilli in Sputum Liquified by Pyridine.** Marthe Giraud and E. Derrien, Soc. de Biol., Nov. 18, 1916, recommend the following procedure for tenacious sputum: Add 15 c.c. of pyridine to 10 c.c. of sputum, mix thoroughly and let stand 5-10 minutes till liquification takes place, although no harm results from longer contact. Centrifuge and examine the sediment as usual. They claim that this method is less "brutal" than that by hypochlorite and heat. (By the way, the word brutal in French, is sometimes used for various experiments, especially on animals in a way that may mislead kind-hearted persons. It applies to the scientific side of an experiment and means crude, or lacking in delicacy).

